# Modeling tools for dengue risk mapping - a systematic review

**DOI:** 10.1186/1476-072X-13-50

**Published:** 2014-12-09

**Authors:** Valérie R Louis, Revati Phalkey, Olaf Horstick, Pitcha Ratanawong, Annelies Wilder-Smith, Yesim Tozan, Peter Dambach

**Affiliations:** Institute of Public Health, Heidelberg University Medical School, Heidelberg, Germany; Division of Epidemiology and Public Health, Nottingham Medical School, University of Nottingham, Nottingham, UK; Department of Public Health and Clinical Medicine, Epidemiology and Global Health, Umeå University, Umeå, Sweden; Lee Kong Chian School of Medicine, Nanyang Technological University, Singapore, Singapore; Department of Nutrition, Food Studies and Public Health, Steinhardt School of Culture, Education and Human Development, New York University, New York, NY USA

**Keywords:** Dengue, Systematic review, Risk mapping, Prediction, Surveillance, Dengue control, Remote sensing, GIS, Spatial, Land cover

## Abstract

**Introduction:**

The global spread and the increased frequency and magnitude of epidemic dengue in the last 50 years underscore the urgent need for effective tools for surveillance, prevention, and control. This review aims at providing a systematic overview of what predictors are critical and which spatial and spatio-temporal modeling approaches are useful in generating risk maps for dengue.

**Methods:**

A systematic search was undertaken, using the PubMed, Web of Science, WHOLIS, Centers for Disease Control and Prevention (CDC) and OvidSP databases for published citations, without language or time restrictions. A manual search of the titles and abstracts was carried out using predefined criteria, notably the inclusion of dengue cases. Data were extracted for pre-identified variables, including the type of predictors and the type of modeling approach used for risk mapping.

**Results:**

A wide variety of both predictors and modeling approaches was used to create dengue risk maps. No specific patterns could be identified in the combination of predictors or models across studies. The most important and commonly used predictors for the category of demographic and socio-economic variables were age, gender, education, housing conditions and level of income. Among environmental variables, precipitation and air temperature were often significant predictors. Remote sensing provided a source of varied land cover data that could act as a proxy for other predictor categories. Descriptive maps showing dengue case hotspots were useful for identifying high-risk areas. Predictive maps based on more complex methodology facilitated advanced data analysis and visualization, but their applicability in public health contexts remains to be established.

**Conclusions:**

The majority of available dengue risk maps was descriptive and based on retrospective data. Availability of resources, feasibility of acquisition, quality of data, alongside available technical expertise, determines the accuracy of dengue risk maps and their applicability to the field of public health. A large number of unknowns, including effective entomological predictors, genetic diversity of circulating viruses, population serological profile, and human mobility, continue to pose challenges and to limit the ability to produce accurate and effective risk maps, and fail to support the development of early warning systems.

**Electronic supplementary material:**

The online version of this article (doi:10.1186/1476-072X-13-50) contains supplementary material, which is available to authorized users.

## Background

Dengue is an arboviral disease, transmitted by two main vectors, *Aedes aegypti* and *Ae. albopictus*. It is found mostly in the tropical and subtropical regions of Asia and South America, and its transmission is influenced by manifold factors, including vector mosquito density, circulating virus serotypes, and the susceptibility of human populations. It is estimated that the annual number of dengue infections lies between 50 and 100 million, with about 25,000 deaths worldwide in 2013 [1]. Demographic change, urbanization, inadequate domestic water supplies, migration [2], and introduction to new areas via international travel have led to an increase in the global incidence of dengue and about 3.6 billion people currently at risk [3]. Additionally, thespread and establishment of dengue is facilitated by a changing climate [4, 5].

Because of a changing environment and population immunological profile, dengue transmission and disease show inherently dynamic spatial and temporal patterns. The non-homogeneous character and instability in the distribution of potentially influential predictors make it difficult to ascribe epidemiological changes to single factors. Despite the complexities, an analysis of the variables linked to the distribution of vectors and identified dengue cases can be a useful tool to generate spatial and temporal scenarios for dengue [6].

Surveillance tools, such as incidence maps, have been utilized to enhance public health preparedness for dengue outbreaks by providing a visual aids for reaching a decision. Although plotted disease occurrence can to some extents facilitate the allocation of public health resources, there is an urgent need for methods that will allow an assessment of the dengue epidemiology over time and space. Such tools have been developed and have gained importance in the last decade, but most of them were not used in the public health context because of their complexity and the extensive need for input data. With this systematic review we attempt to address the research gap caused by the lack of structured overviews of available methods, of relevant predictors and of types of dengue risk maps. We provide a comparison of the most important predictors and the most commonly used modeling methods in order to generate specific types of risk maps, which may have different applicability and relevance for public health decisions. Increased availability of dengue surveillance and prediction maps would allow public health workers to identify and target high- risk areas with appropriate and timely control measures.

The objectives of this systematic review were:

- to collect and describe currently available dengue risk maps,
- to assess the underlying modeling predictors used for developing dengue risk maps,
- to describe methods used for dengue risk mapping and discuss their applicability in the context of public health.

## Methods

### Search terms and databases

This review follows the guidelines for systematic reviews and meta-analyses as laid out in the PRISMA statement [7]. It was carried out between October 2013 and January 2014. All data were extracted by two independent researchers, and discrepancies were resolved concordantly. The following databases were searched electronically: PubMed, Web of Science, World Health Organization (WHOLIS), Centers for Disease Control and Prevention (CDC) and OvidSP. In the first step of the multi-level approach we used MeSH (Medical Subject Heading) terms (for PubMed) and undertook plain text searches for keywords connected with Boolean operators. Searches were run for 10 different combinations of keywords derived from the following categories: i) dengue, ii) geographic tools (e.g. remote sensing, GIS, mapping) iii) surveillance and monitoring, and iv) spatial and spatio-temporal models and cluster analysis. The databases of WHOLIS and the WHO regional databases were searched for grey literature, and we manually searched the reference lists found in the electronic search. No restrictions on time period or language of publication were applied. Although the search was conducted in English language only, no articles were excluded from full text assessment if published in another language.

**Figure 1:**
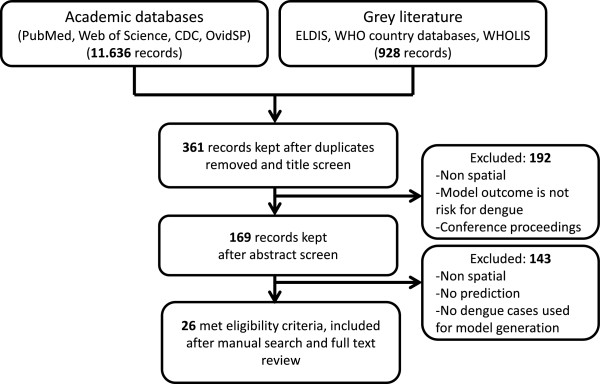
**Flow diagram of article selection and inclusion/exclusion process.**

Results were combined using Zotero (http://zotero.org), and duplicates were removed during a second round of revisions. Once the search results were obtained, the selection of studies for inclusion was made using a two-stage approach. During the first stage, two independent researchers selected articles from the search results based on titles and abstracts, excluding those deemed irrelevant to the topic. Disagreements were resolved with mutual consent. The bibliographies of reviewed articles were scanned for additional literature. Studies relevant to the research questions were assessed for full text, including those studies for which inclusion was uncertain on the basis of title or abstract screen. All articles retained after the first stage went through a full-text review performed independently by two reviewers (Figure 1).

### Inclusion and exclusion criteria

The following inclusion criteria were used: (i) Full text articles that generate risk maps for dengue occurrence. Risk maps were defined as maps obtained through some modeling approach with the purpose of quantifying the risk of dengue over a geographical area; (ii) studies that use dengue morbidity or mortality and at least one additional predictor for model generation. The following exclusion criteria were applied: (i) models dealing only with a temporal component of dengue risk; (ii) studies dealing exclusively with dengue in international travelers; and (iii) abstracts, letters, newspaper articles and lectures representing single expert opinions.

### Data extraction

All data were extracted and collected manually in a matrix. Collected variables included—besides general information—the type of study, the data collection period and the prediction models used. Other analyzed characteristics included the scale of the study (e.g. district or a whole country), thespatial resolution (e.g. at household level or aggregated to municipalities or larger areas), and study areas were distinguished on the basis of their urban or rural characteristics. Detailed information was extracted about variables incorporated in the generation of the risk maps. Besides dengue morbidity and mortality data, which were a prerequisite, entomological data (adult vectors, mosquito larvae), and socio-economic, climatic, and environmental predictors were extracted. The category of “modeled risk” distinguishes between a risk map for dengue and the probability of dengue vector occurrence. Finally the study output regarding the type of risk maps and its relevance was analyzed, along with the key findings. The key findings section presents suggested correlations between independent variables and dengue occurrence. Modeling variables were summarized in a narrative description of each study (Additional file 1: Table S1).

## Results

### Summary of observations

#### General characteristics of the selected studies

Twenty six citations met the eligibility criteria and were included in this review (Figure 1). The selected references are indicated in round brackets ( ) and listed in the Table 1. Details of all the studies are included in Additional file 1: Table S1 and analyzed in the sections that follows. Although no time period restriction was applied in the search, none of the 361 records screened for titles was published before 1992, and all selected articles were published in the last 10 years (2005 onwards). Two thirds of the selected studies were published in the last four years.

**Table 1:** **List of publications selected for the systematic review**

ID	Selected studies
(1)	S. Arboleda, N. Jaramillo-O, and A. T. Peterson, “Mapping environmental dimensions of dengue fever transmission risk in the Aburrá Valley, Colombia,” Int. J. Environ. Res. Public. Health, vol. 6, no. 12, pp. 3040–3055, Dec. 2009.
(2)	K. C. Castillo, B. Koerbl, A. Stewart, J. F. Gonzalez, and F. Ponce, “Application of spatial analysis to the examination of dengue fever in Guayaquil, Ecuador,” Spat. Stat. 2011 Mapp. Glob. Change, vol. 7, pp. 188–193, 2011.
(3)	R. Cordeiro, M. R. Donalisio, V. R. Andrade, A. C. N. Mafra, L. B. Nucci, J. C. Brown, and C. Stephan, “Spatial distribution of the risk of dengue fever in southeast Brazil, 2006-2007,” BMC Public Health, vol. 11, p. 355, 2011.
(4)	M. C. de Mattos Almeida, W. T. Caiaffa, R. M. Assunção, and F. A. Proietti, “Spatial vulnerability to dengue in a Brazilian urban area during a 7-year surveillance,” J. Urban Health Bull. N. Y. Acad. Med., vol. 84, no. 3, pp. 334–345, May 2007.
(5)	D. P. O. de Melo, L. R. Scherrer, and Á. E. Eiras, “Dengue fever occurrence and vector detection by larval survey, ovitrap and MosquiTRAP: a space-time clusters analysis,” PloS One, vol. 7, no. 7, p. e42125, 2012.
(6)	S. K. Dickin, C. J. Schuster-Wallace, and S. J. Elliott, “Developing a Vulnerability Mapping Methodology: Applying the Water-Associated Disease Index to Dengue in Malaysia,” Plos One, vol. 8, no. 5, May 2013.
(7)	R. F. Flauzino, R. Souza-Santos, C. Barcelllos, R. Gracie, M. de A. F. M. Magalhães, and R. M. de Oliveira, “Spatial heterogeneity of dengue fever in local studies, City of Niterói, Southeastern Brazil,” Rev. Saúde Pública, vol. 43, no. 6, pp. 1035–1043, Dec. 2009.
(8)	B. Galli and F. Chiaravalloti Neto, “(Temporal-spatial risk model to identify areas at high-risk for occurrence of dengue fever),” Rev. Saúde Pública, vol. 42, no. 4, pp. 656–663, Aug. 2008.
(9)	H. Hassan, S. Shohaimi, and N. R. Hashim, “Risk mapping of dengue in Selangor and Kuala Lumpur, Malaysia,” Geospatial Health, vol. 7, no. 1, pp. 21–25, Nov. 2012.
(10)	N. A. Honório, R. M. R. Nogueira, C. T. Codeço, M. S. Carvalho, O. G. Cruz, M. de A. F. M. Magalhães, J. M. G. de Araújo, E. S. M. de Araújo, M. Q. Gomes, L. S. Pinheiro, C. da Silva Pinel, and R. Lourenço-de-Oliveira, “Spatial evaluation and modeling of Dengue seroprevalence and vector density in Rio de Janeiro, Brazil,” PLoS Negl. Trop. Dis., vol. 3, no. 11, p. e545, 2009.
(11)	W. Hu, A. Clements, G. Williams, S. Tong, and K. Mengersen, “Spatial patterns and socioecological drivers of dengue fever transmission in Queensland, Australia,” Environ. Health Perspect., vol. 120, no. 2, pp. 260–266, Feb. 2012.
(12)	P. Jeefoo, N. K. Tripathi, and M. Souris, “Spatio-temporal diffusion pattern and hotspot detection of dengue in Chachoengsao province, Thailand,” Int. J. Environ. Res. Public. Health, vol. 8, no. 1, pp. 51–74, Jan. 2011.
(13)	H. M. Khormi and L. Kumar, “Modeling dengue fever risk based on socioeconomic parameters, nationality and age groups: GIS and remote sensing based case study,” Sci. Total Environ., vol. 409, no. 22, pp. 4713–4719, Oct. 2011.
(14)	H. M. Khormi, L. Kumar, and R. A. Elzahrany, “Modeling spatio-temporal risk changes in the incidence of Dengue fever in Saudi Arabia: a geographical information system case study,” Geospatial Health, vol. 6, no. 1, pp. 77–84, Nov. 2011.
(15)	H. M. Khormi and L. Kumar, “Assessing the risk for dengue fever based on socioeconomic and environmental variables in a geographical information system environment,” Geospatial Health, vol. 6, no. 2, pp. 171–176, May 2012.
(16)	R. Lowe, T. C. Bailey, D. B. Stephenson, R. J. Graham, C. A. S. Coelho, M. S. Carvalho, and C. Barcellos, “Spatio-temporal modelling of climate-sensitive disease risk: Towards an early warning system for dengue in Brazil,” Comput. Geosci., vol. 37, no. 3, pp. 371–381, Mar. 2011.
(17)	E. A. Machado-Machado, “Empirical mapping of suitability to dengue fever in Mexico using species distribution modeling,” Appl. Geogr., vol. 33, no. 1, pp. 82–93, Apr. 2012.
(18)	A. T. Peterson, C. Martínez-Campos, Y. Nakazawa, and E. Martínez-Meyer, “Time-specific ecological niche modeling predicts spatial dynamics of vector insects and human dengue cases,” Trans. R. Soc. Trop. Med. Hyg., vol. 99, no. 9, pp. 647–655, Sep. 2005.
(19)	X. Porcasi, C. H. Rotela, M. V. Introini, N. Frutos, S. Lanfri, G. Peralta, E. A. De Elia, M. A. Lanfri, and C. M. Scavuzzo, “An operative dengue risk stratification system in Argentina based on geospatial technology,” Geospatial Health, vol. 6, no. 3, pp. S31–42, Sep. 2012.
(20)	C. Rotela, F. Fouque, M. Lamfri, P. Sabatier, V. Introini, M. Zaidenberg, and C. Scavuzzo, “Space-time analysis of the dengue spreading dynamics in the 2004 Tartagal outbreak, Northern Argentina,” Acta Trop., vol. 103, no. 1, pp. 1–13, Jul. 2007.
(21)	A. Shafie, “Evaluation of the Spatial Risk Factors for High Incidence of Dengue Fever and Dengue Hemorrhagic Fever Using GIS Application,” Sains Malays., vol. 40, no. 8, pp. 937–943, Aug. 2011.
(22)	M. Sriprom, K. Chalvet-Monfray, T. Chaimane, K. Vongsawat, and D. J. Bicout, “Monthly district level risk of dengue occurrences in Sakon Nakhon Province, Thailand,” Sci. Total Environ., vol. 408, no. 22, pp. 5521–5528, Oct. 2010.
(23)	T.-H. Wen, N. H. Lin, C.-H. Lin, C.-C. King, and M.-D. Su, “Spatial mapping of temporal risk characteristics to improve environmental health risk identification: a case study of a dengue epidemic in Taiwan,” Sci. Total Environ., vol. 367, no. 2–3, pp. 631–640, Aug. 2006.
(24)	T.-H. Wen, N. H. Lin, D.-Y. Chao, K.-P. Hwang, C.-C. Kan, K. C.-M. Lin, J. T.-S. Wu, S. Y.-J. Huang, I.-C. Fan, and C.-C. King, “Spatial-temporal patterns of dengue in areas at risk of dengue hemorrhagic fever in Kaohsiung, Taiwan, 2002,” Int. J. Infect. Dis. IJID Off. Publ. Int. Soc. Infect. Dis., vol. 14, no. 4, pp. e334–343, Apr. 2010.
(25)	P.-C. Wu, J.-G. Lay, H.-R. Guo, C.-Y. Lin, S.-C. Lung, and H.-J. Su, “Higher temperature and urbanization affect the spatial patterns of dengue fever transmission in subtropical Taiwan,” Sci. Total Environ., vol. 407, no. 7, pp. 2224–2233, Mar. 2009.
(26)	H.-L. Yu, S.-J. Yang, H.-J. Yen, and G. Christakos, “A spatio-temporal climate-based model of early dengue fever warning in southern Taiwan,” Stoch. Environ. Res. Risk Assess., vol. 25, no. 4, pp. 485–494, May 2011.

#### Scale and scope

**Figure 2:**
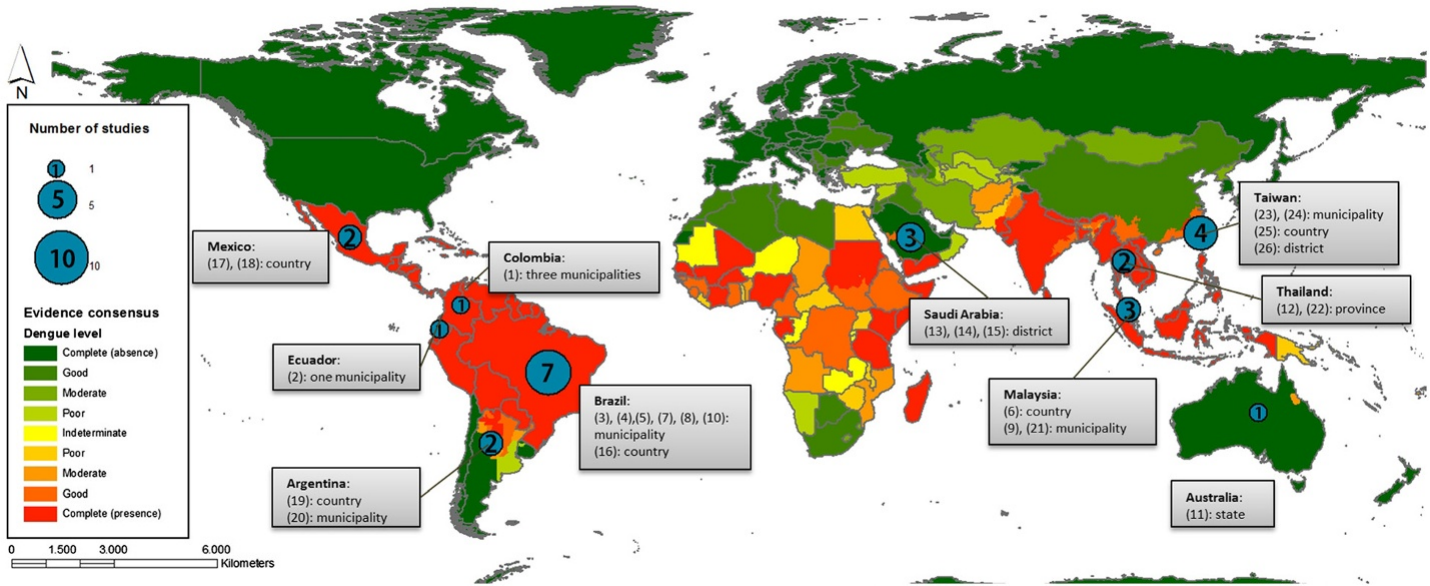
**World map of dengue evidence consensus (adapted from Brady et al. [**[8]**]) with number of publications reviewed in respective countries.** Geographic scale (municipality, district, state/province, country) of studies is given in grey boxes.

The distribution of studies by region, country and administrative level is shown in Figure 2. Most of the studies undertaken at local scales took place in urban areas, known to be at high risk for dengue transmission [1].

Because they were more general and geographically extended, studies done at country, state and province levels were more likely to include rural areas.

#### Study design

**Figure 3:**
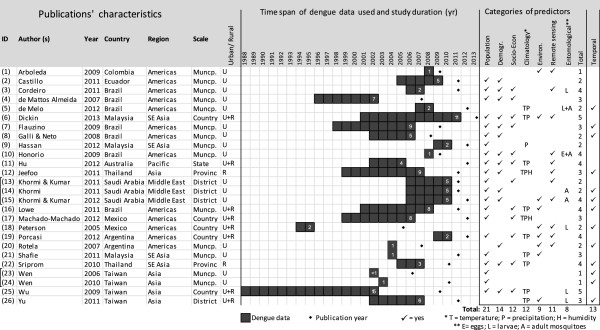
**Characteristics of reviewed articles indicating reference ID, geographical origin, time span, predictor categories used for risk mapping (i.e.; population, demographic, socio-economic, climatological, environmental (T = temperature; P = precipitation), entomological data (E = eggs, L = larvae, A = adult mosquitoes)) with total number tallied, remote sensing, and temporal component.** Brackets indicate a common working group.

All studies were retrospective. Most of them used secondary data on dengue cases from surveys implemented in the health surveillance systems, although several studies (3, 10, 19, 20, 23, 24) ran over a shorter period of time during occurring epidemics. Besides reported dengue cases, critical predictors used for model generation and dengue risk maps included variables from a variety of categories, including population, demography, socioeconomic status, climate, environment, and entomology. Reviewed articles used dengue data ranging from one to 15 years, with an average and median of five years (Figure 3). Eleven studies used dengue data sets shorter than two years while 13 of the 26 studies used data sets of five years or longer duration.

### Predictors

#### Population, demographic and socioeconomic data

Most studies (21 out of 26) used population distribution and density to describe and model the risk of dengue occurrence (Figure 3). In general these data originated from national censuses, but the working group in Saudi Arabia (13) used satellite imagery to estimate population density. Demographic data, primarily age, was used in 14 studies. Social predictors, such as educational level, occupational status, and income, as well as housing conditions, were used to assess environmental conditions and hygiene. Educational level and type of employment were often used as proxies for wealth and awareness of dengue and its transmission (3, 4, 10, 11). Furthermore, housing conditions, which determined in many cases the type and amount of vector breeding in the vicinity, were used in eight studies (3, 4, 6, 7, 11, 17, 22). Less commonly used predictors included household water delivery, whether or not respondents spent time outside their neighborhood, pronounced presence of garbage, reports of mosquito bites during the day (3); proportion of children under 10 and woman over 64 years, ratio between number of commercial establishments and number of households in census tracts (4); household overcrowding, aborigine origin, elders living alone, disability, and numbers of clinics (25). The most commonly used predictors included age, gender, education level, and income (e.g. 4, 12, 22). Most studies that included socio-economic predictors identified some of them as significant.

#### Climatic data

Half of the studies incorporated climatic predictors in their model generation. Predominantly used predictors were precipitation and air temperature (5, 6, 9, 11, 12, 15–17, 19, 22, 25, 26) while two studies used relative humidity (12, 17). These climatic variables were used to describe both spatial and temporal risk of dengue transmission. Examples of climatic predictors that were included in the reviewed models were “minimum temperature of the coldest month” (17), lagged minimum and maximum temperatures (26), and average monthly rainfall and average monthly maximum temperature (11). Two studies (16, 26) used indicators associated with the El-Niño-Southern-Oscillation (ENSO) as predictors.

#### Environmental data

Environmental information comprised data on vegetation, surface water and land cover. Those predictors were mostly used to describe the suitability of environmental conditions for vector breeding and survival. These types of data can be either registered in the field, or derived via remote sensing, especially on larger scales. As a result, out of seven studies (1, 6, 15, 18–21) only one (21) did not collect environmental information partially or completely via remote sensing. Vegetation as a proxy for surface water, mostly detected via the commonly used Normalized Difference Vegetation Index (NDVI), and the direct detection of water provided an opportunity to detect vector breeding sites.

#### Remote sensing

Remote sensing in itself is not associated with specific types of variables but it is a tool to obtain various types of data, typically through the use and analysis of satellite imagery. Eleven studies incorporated remotely sensed data (Figure 3). The purposes of these studies were diverse, and a variety of data were acquired. While three studies (6, 10, 12) used remote sensing for map creation or to obtain a land cover map, six studies (1, 13, 15, 18–20) used satellite images to gain information on the environmental vector breeding appropriateness (NDVI, surface water, elevation, slope), or anthropo-geographic factors (population density, neighborhood quality). Two studies (11, 19) used remotely sensed data on climatic variables to address the lack of routinely collected data from meteorological stations. While high-resolution imagery was used for socioeconomic variables (13, 15, 19), environmental predictors were derived at a much coarser scale, using Landsat (1, 20), ASTER (19), and AVHRR (18) satellite imagery.

#### Entomological data

Entomological data were collected at egg, larval and adult stages. Mosquito egg and larval collections were done either with ovitraps, classical dipping techniques, or recording of an index, such as Breteau index, which is defined as the number of containers positive for *Aedes* larvae per 100 houses inspected. Six studies (3, 5, 10, 18, 25, 26) used egg or larval counts as input predictors for model generation. Capture activities used different types of traps (light traps, CO_2_ traps) to estimate adult mosquito population densities. Four studies (5, 10, 14, 15) sampled the adult vector mosquitoes. Entomological predictors were used for model development mainly at the municipality level, although these predictors were also employed at district and country levels (14, 15, 18, 25). Only two studies (5, 10) used both larval and adult mosquito data, both at the municipality level. Among the eight studies that included an entomological survey, three found that neither larval data (5, 25) nor mosquito abundance data (10) were associated with dengue data. In contrast three studies (3, 18, 26) included larval data as significant variables for higher dengue risk while a study (5) found both eggs and adult mosquitoes associated with dengue cases. Two studies (14, 15) found that dengue hotspots corresponded to adult mosquito presence but not necessarily to the highest vector abundance.

#### Temporal component

Out of 26 studies, 13 dealt with temporal model components added onto spatial risk maps. The temporal classification of dengue risk was not homogeneous. Approaches differed from long-term predictions under the influence of climate change (e.g. the introduction of dengue to new areas, over mid-term phenomena inter-annual differences, such as *El Niño*) to intra-annual particularities, such as seasonality. To elicit more precise temporal predictions or pattern recognitions, meteorological predictors of higher temporal resolution become necessary to accommodate for rapid changes in environmental conditions. Eight studies (5, 6, 12, 15, 16, 18, 22, 26) that included temporal components used weather predictors. Some studies did not use climatic predictors for their temporal models (14, 20, 23, 24) while others incorporated climatic data but did not model temporal risk (9, 11, 19, 21, 25).In summary, among the studies reviewed, the type and number of predictors used varied greatly. Out of the six categories identified (population, demographic, socio-economic, climatic, environmental, and entomological data) (Figure 3), up to five different ones were used in a single model to generate dengue risk maps. Three used only one category in addition to reported dengue cases, namely environmental data (1) and population (23, 24). Most studies used three to four categories while two studies (6, 25) used five different categories. No specific patterns were recognized in the combination of categories across studies.

### Modeling approaches

A variety of mathematical and statistical methods were used to generate dengue risk maps (Table 2). Most studies used multiple or complementary approaches. Reported dengue cases were used along with other selected predictors (socioeconomic, climatic and demographic factors) to estimate the risk of dengue occurrence over a geographical area. The maps were based on values computed from the selected predictors for each map pixel (smallest surface area with a specific value). Methodologically, we distinguish the use of models from that of indices as such a way as to obtain risk estimates. Models imply individual use of variables while indices use a composite of variables computed from the available data.

**Table 2:** **Overview of modeling approaches used in reviewed publications**

Publications ID→	1	2	3	4	5	6	7	8	9	10	11	12	13	14	15	16	17	18	19	20	21	22	23	24	25	26	Total
***Types of methods (models or indices)***																											
**Spatial analysis of case clusters/hotspots**		✓			✓		✓	✓		✓		✓		✓	✓					✓						✓	**10**
**Spatial autocorrelation measures**		✓			✓							✓		✓	✓					✓							**6**
**Logistic regression and multinomial models**			✓	✓															✓		✓				✓	✓	**6**
**Generalized Linear Models/General additive model (GAM)**			✓							✓	✓					✓						✓					**5**
**Kernel estimation**							✓					✓													✓	✓	**4**
**Environmental niche modeling/Species distribution modeling (Suitability)**	✓																✓	✓									**3**
**Maximum Entropy (MaxEnt)**	✓																✓		✓							✓	**4**
**Geographically weighted regression**													✓														**1**
**Kriging and co-kriging**									✓																		**1**
**Knox test concept (space- & time-distance)**																				✓							**1**
**Temporal indices (occurrence, duration, intensity)**								✓															✓	✓			**3**
**Water-associated disease index -WADI (Vulnerability)**						✓																					**1**
***Types of risk level***																											
**Categorical risk level**		✓		✓				✓				✓		✓	✓	✓					✓	✓	✓	✓	✓		**12**
**Continous risk level**	✓		✓		✓	✓	✓		✓	✓	✓	✓	✓				✓	✓	✓	✓		✓	✓	✓		✓	**18**
***Types of maps***																											
**Descriptive maps**		✓	✓	✓	✓		✓	✓	✓	✓	✓	✓	✓	✓	✓	✓					✓	✓	✓	✓	✓		**19**
**Validated maps**	✓					✓	✓									✓	✓	✓	✓						✓	✓	**9**
**Predictive maps**	✓					✓					✓					✓	✓	✓	✓	✓						✓	**9**
**Early warning system (EWS)**											✓					✓										✓	**3**

#### Models

Spatial analysis aiming at detecting dengue clusters and hotspots was the most common approach used in 10 studies to generate risk maps; six of which used measures of spatial autocorrelation (Table 2). Cluster detection allowed the identification of areas where dengue cases were concentrated rather than being geographically randomly distributed. Through modeling these areas could be identified as hotspots that are most likely to require public health awareness and intervention. Spatial autocorrelation estimated the degree to which observations close to each other were more likely to be correlated.

Logistic regression models, multinomial models and the more general generalized linear models and general additive models (GAM) were common approaches used to compute risk levels and create maps. One study (26) used a generalized linear mixed model (GLMM), a model that, in addition to the fixed effect, includes a random effect for which the hypothesis of independence of observations is no longer assumed. Environmental niche or species distribution modeling that models the suitability of an environment in this case to dengue cases was used in three studies (1, 17, 18). This approach is commonly used in Ecology to determine where species are more likely to be found or to establish themselves. For studies (1) and (17), the method was based on maximum entropy using MaxEnt algorithm while study (18) adapted a genetic algorithm. Maximum entropy approach was also used in studies (1) and (26) using different computational methods. Because niche modelling and maximum entropy approach can encompass large area and diverse environmental conditions, these categories of models are also well suited for larger scales, e.g. country scale (19). Kernel estimations were used in four studies (7, 12, 25, 26). Some methods were found only once, namely geographically weighted regression (13), kriging and co-kriging (9), and Knox test concept to generate space-distance and time-distance aimed at identifying spatio-temporal clusters (20).

#### Indices

Indices were used in four studies. One approach consisted of using three different temporal indices that describe occurrence, duration and intensity of dengue based on reported dengue cases and population data. It was developed in Taiwan by Wen et al. 2006 (23), further elaborated in 2010 (24), 2010, and applied by Galli & Netto in Brazil (2008) (8). This use of indices was simpler to implement because it did not necessitate further computation. Another approach employed only by one group (6) consisted of using the Water Associated Disease Index (WADI) and aimed to model vulnerability to different water-associated diseases, including dengue.

#### Risk level

Risk levels computed from models or indices are usually continuous estimates ranging from zero to one, with “zero” indicating no risk of dengue and “one” confirmed dengue in the area. Eighteen studies displayed raw continuous values while twelve categorized the risk level, alone or in addition to continuous values (Table 2). The cut-off values used for categorization were rarely given explicitly. The numbers of risk categories ranged from three to five in a gradation from low to high. Studies using the temporal indices (8, 23, 24) used more complex categorizations that included eight levels and incorporated a temporal component. Three studies (2, 12, 24) computed the categorized risk values of a given area (i.e. map pixel) by considering the continuous risk value of the area itself and that of its neighbors, in an analysis with Local Indicators of Spatial Autocorrelation (LISA). Study (24) combined the temporal indices approach and applied LISA methods to generate a risk map that can be interpreted in a conventional “low to high” gradation.

**Figure 4:**
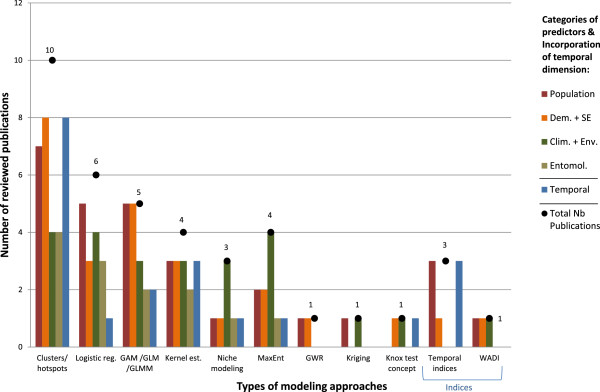
**Types of modeling approaches vs. types of predictors (temporal, population, demographic and socio-economic (Dem. + SE), climatological and environmental (Clim. + Env), and entomological (Entomol.) used in reviewed publications.** Abbreviations: reg. = regression; GAM/GLM/GLMM = general additive model/generalized linear models/generalized linear models; MaxEnt = Maximum Entropy; GWR = geographically weighted regression; est. = estimation; WADI = water-associated disease index.

In summary, the reviewed publications modeling approaches could be grouped in four categories in view of the different types of predictors used, namely: i) population, ii) demographic and socioeconomic, iii) climatic and environmental, and iv) entomological (Figure 4 and Additional file 2: Table S2). A fifth category was added to indicate a temporal component. In modeling approaches used by more than one study, all five categories of predictors were included (Figure 4). However, this was not the case in modeling approaches used only by a single study. As a consequence, patterns regarding types of predictors as a function of methodology were not easily discernable. In general modeling approaches used only in one study used fewer predictor categories. Those based on indices used a maximum of three categories that always included population and socio-economic predictors. The small number of such studies, however, may not reflect the full potential and variety of possible applications.

### Types of risk maps

We divided the reviewed risk maps into four different categories; descriptive, validated, predictive and early warning system (EWS). Within this review, 19 studies out of 26 included descriptive maps (Table 2). For the most part, these maps described retrospective dengue occurrence within a context of specific socio-economic or environmental conditions. Although classified as descriptive, they were not simply inventory maps directly plotting raw databut presented the results of a modeling approach. They were nonetheless classified as descriptive because the resulting risk maps were mostly descriptive and lacked validation and predictive values. They were primarily based on the premise that the location of previous events was a good indicator of where future events might occur.

**Figure 5:**
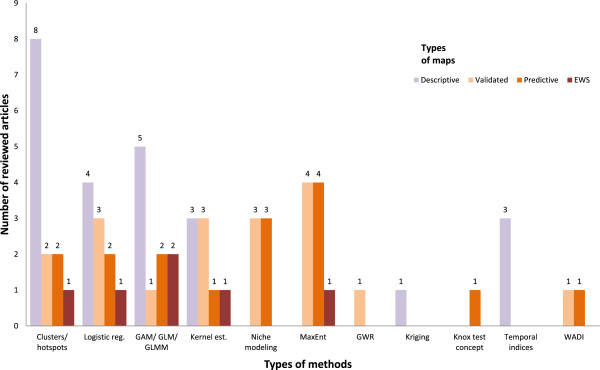
**Types of modeling approaches vs. types of maps.** Abbreviations: EWS: Early warning system, reg. = regression; GAM/GLM/GLMM = general additive model/generalized linear models7/generalized linear models; MaxEnt = Maximum Entropy; GWR = geographically weighted regression; est. = estimation; WADI = water-associated disease index.

Nine of the reviewed studies (1, 6, 7, 16–19, 25, 26) were classified as including validated maps. They included an evaluation of the model by comparing predicted to observed values to assess the quality of the model and the goodness of fit. This approach is usually a step toward making predictive maps. Nine of the 26 studies included such predictive maps. They aimed to model dengue occurrence in areas where data were not readily available by using knowledge on previous occurrence, socio-economic or environmental characteristics. Data sets were typically subdivided into training and test sets to allow for simulation of area without known data and subsequently test the quality and fit of the model. Only three studies (11, 16, 26) made an attempt at integrating an early warning system (EWS) and they all included advanced approaches and a temporal component. The approaches with EWS attempted to establish criteria in early recognition of disease outbreaks for application in public health.Considering the types of maps created in relation to the modeling approaches used (Figure 5), it appears that the majority of descriptive maps used a cluster analysis and hotspot detection approaches. As these approaches were also used for all other types of maps it represented an entry point in creating risk maps. Logistic regressions, general additive models, generalized linear models, and kernel estimations are general approaches that are widely used for the creation of all types of maps. Niche modeling and maximum entropy approaches allowed the generation of validated and predictive maps. The method using temporal indices represented a simple approach that was useful to create descriptive maps. Generating maps that include EWS relied on a variety of modeling approaches of higher complexity.

## Discussion

### Predictors

A large variety of predictors were used to create dengue risk maps and there was no pattern of predictor use associated with specific approaches.

#### Population, demographic and socioeconomic data

Demographic data were mainly used by studies that aimed to develop risk maps at a local scale, such as wards or towns. Socioeconomic and demographic data were used as a proxy for population mobility and housing conditions [9] and potential exposure to the vector. In this context, these predictors are known to be associated with awareness about dengue, the use of personal protective measures, and health seeking behavior [10]. In the publications reviewed here, predictors associated with poor housing conditions were reliable indicators for elevated dengue incidence (3, 4, 6, 7, 11, 17, 22), regardless of whether the data were collected by remote sensing or by other means. A number of maps were limited to describing where dengue cases had occurred and employed approaches that were mostly based on population data (9, 13). However, such descriptive maps were useful for visualizingdisease hotspots and areas of heightened dengue risk at a given time and space (5, 7, 10, 12, 14, 15). They were also effective at identifying unusual variables that might be important for dengue risk. For example study (21) identified, among others, proximity to cemetery, hospital, schools and public infrastructure as significant predictors; three studies (4, 12, 22) identified a combination of gender and specific age groups.

#### Climatic and environmental data – temporal component

Climatic and weather data were found to be particularly useful for the generation of predictive risk maps and almost all the reviewed publications that have a temporal component include such data. These findings show that high resolution for this type of data is a prerequisite for the creation of predictive maps, especially those used for early warning. Although purely temporal modeling of dengue was shown to produce good forecasts of the general dengue situation in some studies [11], our review showed that for the generation of maps at higher resolution, other factors, such as human movements or housing conditions, are more likely to be linked to the occurrence of dengue cases.

#### Remote sensing

Although remotely-sensed data are not used to detect *Aedes* breeding sites directly, the information on the type of land cover allowed an indirect assessment of appropriateness for vector breeding and survival (1, 6, 15, 18–21) [12]. Remote sensing was found to be an efficient tool to collect data on different predictors over large areas and should be considered for inclusion in future models for risk map creation. In contrast to the fields of malaria and other vector borne infectious diseases, our systematic literature review showed that reliable predictors for dengue have not yet been established. Unlike the situation with malaria where the vector often breeds in meter-size ponds, a major problem with dengue is the high number of non-detectable breeding sites [13–16]. The studies reviewed here did not address this issue. With advances in satellite technology the resolution of spatial imagery is likely to increase, allowing the estimation of additional predictors to appraise where humans and vectors interact. Information extracted from satellite imagery can be seen as a cost-effective approach to collecting important data for the creation of dengue prediction maps and the steering of control strategies. The wide variety of predictors sourced via remote sensing can provide an underlying “base map” displaying human and natural prerequisites for dengue transmission. Making them available for public health institutions would be useful in reducing the need for extensive data searches and in limiting the costs for risk map creation [17].

#### Entomological data

The dengue vector *Aedes* is a prerequisite for dengue transmission. However, despite the intensive use of entomological data in several studies (3, 5, 10, 15, 18, 25, 26), establishing the link between entomological aspects measured in the field (larvae abundance, mosquito capture, ovi-trapping) and clinical dengue cases remains elusive to this day. Some studies identified a positive correlation between larval abundance and dengue case location (14) while others found no link (5, 10). Studies that included entomological predictors typically put a high emphasis on those predictors although there were not always statistically significant. These contradictory results indicate that effective larval samplings representative of actual entomological profiles are difficult to achieve and that most surveys yield spurious results that are not useful for prediction. The literature suggests that pupae surveys, using the pupa index (defined as the number of pupae per 100 houses inspected), are better adapted to estimate vector abundance [18]. However, none of the reviewed studies used such pupae surveys as the basis for risk mapping. Adult vector abundance and oviposition showed a more consistent correlation with disease outcomes (5) although medium rather than highest vector abundance may be associated with highest dengue risk (15). Adult mosquito capture was sparsely utilized, possibly because they are more difficult and costly to be put in place and kept up running. Commonly used entomological indices such as house-, container- or Breteau-index were originally designed to check the efficacy of vector control measures rather than evaluate vector density. Pupa and adult indices may provide more reliable predictors but the evidences remain limited. Because entomological surveys are labor-intensive and costly, it would be important to establish their relevance in risk mapping before recommending their use.

#### Research gaps

##### Host serological profile and virus genetic diversity

The weakness of current dengue prediction maps originates from the fact that dengue is highly dynamic and extremely multifactorial. Only a fraction of dengue infections are symptomatic, and host immunity plays an important role in diffusion models. Immunology is difficult to track and requires data on the population´s serology, and poses a major challenge for the generation of current models. Only one study (20) integrated serology profile and virus serotyping. The coexistence of different serotypes and viral lineages adds complexity to modeling attempts [19, 20]. Even when serotypes are documented, they may be insufficient to explain the full spatial heterogeneity of dengue transmission [21]. The association between introduction of a new serotype and outbreaks, and the time lag between the two require further studies using strain specific transmission data [22]. Furthermore viral genetic variability may be an essential component to better understand the transmission dynamics and its spatial patterns. For future studies that include dengue serotyping, those data could be used as an additional predictor for establishing an early warning signal, although it does not always predict the occurrence of an immediate epidemic [23].

#### Mobility

Human mobility and movements seem to have much greater influence than previously thought [24–26], even on a local scale [27] and are not addressed in depth in any of the models reviewed. In our review, predictors dealing with mobility were found only in one study (3): “Staying at home during the day”. Capturing human mobility, which may be expected to be lower in young and older age groups, along with vector movement and working at relevant spatial scale is critical in adequately modeling dengue dynamics [28]. Studies tracking individual mobility patterns using cell phone data at various geographical locations have shown than 90% of the population has regular patterns that can be described by simples rules [29]. It would thus be useful to geo-reference place where people spend most of their time (homes, schools, office places, markets, places of worship, hospitals, etc.) when studying mobility pattern related to dengue cases in a community. This is a reasonable approach to tackle localized dengue outbreaks because there is evidence that most dengue transmissions occur within a small time-space cluster, namely within one kilometer and within one month of an identified case [30]. For this purpose geo-referencing of dengue datasets is necessary. Ubiquitous technology that can achieve such task is nowadays available and do not pose a problem anymore. This will allow the exploitation of data at a much finer scale as recommended for improved surveillance [22]. Encompassing not only the home address of patients but the locations of their usual whereabouts would give a much more accurate picture of the potential infection area. Assuming that ethical considerations are respected and that data are exploited at population level, this could better exploit the potential of maps that are still under-utilized in routine public health applications.

As more sophisticated models are developed to better capture the complexity of dengue dynamics [31], creation of risk maps would benefit from the above mentioned approaches to enhance predictive capability. Approaches using mechanistic models rather than statistical approaches as was the case in the reviewed studies may provide more appropriate tools to explore this field of research including the development of early warning systems.

### Modeling approaches

The modeling approaches used in the reviewed publications were varied and ranged from statistics, geography, ecology to genetics. Cluster detection used for dengue cases and hotspot analysis is often used as a starting point to generate risk maps. Approaches used in statistics and applied to risk mapping included general additive models, generalized linear models, kernel estimation, or Bayesian frameworks. Niche modeling and maximum entropy algorithms are commonly used in ecology. Geographical weighted regression and kriging are a common tool in geography.

#### Models

Approaches based on statistical methodology were very commonly used. Many undertook an analysis of dengue profile in the study area (e.g. 3, 4, 7, 25). A number of them were more complex and mathematically involved, allowing the development of predictive maps. This approach has been used successfully to describe the potential areas of dengue occurrence at a global scale [8]. Several studies successfully used such approaches and usually included a temporal component, as follows: predictive maps (1), spatio-temporal diffusion pattern (12), spatio-temporal model as a precursor for an EWS (16), predictive maps on climate scenarios (17), a genetic algorithm (18), the design and implementation of the dengue risk stratification system at the national and the urban levels (19), predictive incidence maps and spatio-temporal cluster detection (20), and a spatio-temporal EWS (26). Indicators of autocorrelation, used here in six studies (Table 2), are useful to strengthen the models by borrowing information about dengue occurrence from the neighboring or connected areas. This is especially useful when available data is scarce, both at spatial or temporal scale.

Several studies were distinctive in their methodological approaches, and a few employed unique approaches (6, 9, 13, 20). Niche modeling and suitability maps fell in their own category although different methodologies wereused to create the maps: MaxEnt algorithm (1, 17, 19), Bayesian maximum entropy (26), and genetic algorithm (18). There was only one study (19) that demonstrated actual implementation in the field and proved its large-scale applicability. The three studies using temporal indices (8, 23, 24) proposed a practical approach with easy implementation capabilities in a public health context. Study (6) used the water-associated disease index (WADI). This approach was relatively complex, but it was able to generate predictive vulnerability maps encompassing socio-economic and environmental dimensions.

#### Indices

Approaches based on temporal indices (23–24) are simpler and emphasize the temporal component. Although lack the advantage of using spatial data they are useful for visualizing results on maps. In contexts where dengue longitudinal data are reliable but sophisticated analytical spatial analysis is lacking, they may represent a practical public health approach because they are easy to implement using surveillance data collected at district level.

### Types of risk maps

Although all studies used modeling approaches to combine collected influencing predictors, the general nature of maps created in the reviewed studies is diverse. Descriptive, validated, predictive and EWS maps require different predictors and facilitate different public health purposes.

#### Descriptive maps

The majority of 19 studies dealt with descriptive maps, using environmental and human variables associated with dengue cases. One weakness of this type of maps is the limited predictive value, as they only point out past areas of augmented dengue occurrence. Despite their relative simplicity they can help public health authorities in spotting areas that have an increased probability over time and are therefore likely to show similar patterns in future. Those approaches work relatively well in areas where dengue transmission is endemic and the detecting of geographic clusters of cases is of interest. Although predictive capacity is lacking in this case, such information can be valuable to deliver care and interventions such as vector control or vaccination campaigns in the future to areas with high dengue transmission.

#### Validated maps

Validated maps mostly represent a step toward creating predictive maps that are of greater value for public health applications. Predictive maps were heterogeneous in their mode of creation. All nine studies developing predictive maps covered within this review used different sets of predictors for their creation. For the creation of validated maps, most studies used a data subset to test correlations between influencing variables and reported dengue cases. Still lacking the explicit prediction of new dengue cases, those maps were found to permit attribution of elevated risk of case occurrence to specific predictors, such as socioeconomic status and type of neighborhood. Allowing tracing back and spotting specific risk variables is of major importance in a public health context because counter measures can be specifically targeted to eliminate those risk factors, e.g. poor housing conditions, construction sites. In particular, on a local scale such as a neighborhood this type of map provides an excellent planning guide to execute constructional and infrastructural measures. Despite their increased need for input data and hence possibly increased costs, we assess these models as more valuable for this specific use than purely descriptive maps.

#### Predictive maps

Predictive maps (1, 6, 11, 16–20, 26) allow the designation of areas at risk outside the study area where few or no dengue case records are available. Their main strength is to deliver information on a future epidemic situation for a defined point in time and/or an area. Most of the reviewed studies dealing with this type of maps developed models with low spatial resolution and predicted dengue risk on country or state scale. Only two studies (1, 20) were run on municipality level. This fact defines the designated field of use of the maps that become appropriate tools to demarcate risk areas within regions considered to be prone to dengue. On the other hand most of the reviewed studies did not deliver information at a spatial precision that would be sufficient to take actions on a finer scale. Because dengue prediction maps were capable of incorporating climatic variables, available predictive scenarios could range from short-term (e.g.weather) over mid-term (e.g. seasons; ENSO) to long-term (e.g. climate change) influence on dengue epidemiology. From a public health perspective those maps are an ideal tool to prepare larger administrative areas for recurring dengue occurrence or even expected new introduction of dengue due to changes in presence and type of climate, vegetation, population, virus, and vector.

#### Early Warning Systems (EWS)

Early warning systems within this review (11, 16, 26) build up on predictive maps and are therefore included in both of those categories. One study (26) fell short of providing an effective EWS because the model could reproduce only large outbreaks but not smaller ones. Similarly another study (11) had potential for application in EWS, but its applicability was not proven. Study (16) proposed an EWS methodology at a national level covering large administrative divisions, but again its practical applicability remains to be established. Their added value is the capability to deliver more precise forecasts in space and time. All of the reviewed studies developing EWS, incorporated temperature and precipitation in their model building. Those predictors turn out to be crucial for prediction of dengue, mainly because they determine vector breeding and survival and hence disease transmission. EWS are ideal tools to support health system preparedness, allowing optimal resource allocation and steering of interventions in space and time. A recent example is the use of such data to alert public official of potential health risk during a major public event such as the football world cup [32]. Their downside is the high amount of input data needed to build a robust EWS system and the lack of spatial precision.

#### Scale and temporal component: different maps for different purposes

The different types of maps serve different purposes and have different roles in public health applications. A Cochrane review on the evidence on the application of tools for dengue outbreak prediction/detection and trend monitoring in surveillance systems highlighted the lack of evidence about the most feasible and sustainable surveillance [33]. Prospective studies may be needed to better define the most appropriate and most cost-effective dengue surveillance system and trigger for dengue emergency response.

#### Scale

The analyzed studies showed that the more the risk map’s scale moved towards a state or country perspective, the more environmental and climatic factors played a role for their generation. Maps at country and continent scale were found to mostly be suitability maps, evaluating where the environmental conditions are appropriate for vector breeding and survival. This approach is common in studies looking at areas free of dengue but where the virus could potentially spread, given adequate environmental conditions [34]. However, this type of studies was not considered in this review. From a public health point of view, maps at regional or national levels are useful for strategic decisions on how and where to most optimally spend resources [32, 35] while routine surveillance data collected and analyzed at local scale benefit short term response [22]. Acquiring data of good quality at relevant scales is crucial for fostering the development of robust analyses and reliable risk maps [17].

#### Temporal component - applicability and generalizability

For public health applications there is a tradeoff between the complexity of an approach that may be developed in an academic environment and its applicability in day to day applications at a district or national level.

Bridging this gap appears essential for better disease control. As the focus of risk maps shifts from a surveillance system to a system of prediction, the number of driving factors that have to be included into the model will increase. Therefore a tradeoff exists between surveillance approaches that are basically descriptive but easier to implement and predictive approaches that require more data but can generate more valuable information. For some purposes the limited predictive capacities that some surveillance systems offer [36] have to be taken into consideration when deciding for an appropriate tool for use in public health. Given the complexity of spatio-temporal analysis to generate predictive maps, early warning systems for dengue remain difficult. Purely temporal approaches may be more practical for public health routine applications at this stage [11] but research should further develop the field [31] to take advantage of the availability of new technologies and applications that may become mainstream in the future. Only a few studies explicitly mentioned that their methodology could be applied to other diseases (6, 16, 19, 24) even though most approaches were general enough to be applied more widely.

### Study limitations

The search in the literature databases was performed in English language only. Although no language restrictions were applied to the results, literature using keywords and MeSH terms in other languages might have yielded additional relevant studies. Similarly, ambiguous use of technical terms, for example the delimitation between weather, seasonality and climate, and prediction, forecast and risk, within the researched literature could have limited or biased our search results. Most data used for risk mapping are secondary data that have not been collected primarily for the mapping purposes and this may inherently limit the quality of the data analysis. Finally, this review deliberately focused on studies that included dengue cases to link the issues with public health concerns. As a consequence, a number of studies dealing exclusively with entomological risk or with environmental suitability were not considered in this review.

## Conclusions

This review has shown the great diversity of both predictors and modeling approaches employed to create dengue risk maps. There is little standardization and no specific patterns of analysis. This shows that the field of predictive dengue risk mapping is young and still evolving. Dengue is characterized by a high variability of reported clinical cases in time and space. To date, this remains a challenge, and predictive models still lack reliability for anticipating outbreaks. The prediction of spatial and spatio-temporal dengue risk is complex to model and depends on multiple and diverse factors [37, 38]. The distribution and dynamics of dengue transmission are not only contingent on environmental and socioeconomic variables, which most models we surveyed considered. To significantly improve the current ability to describe dengue transmission dynamics, future models need to consider serological profiles, circulating viral serotypes/genotypes and human movements [39]. Geo-referencing data and analyzing data spatially is crucial to achieve this goal. The general availability of mobile devices with geo-referencing abilities makes it possible to speculate that integrating the last two factors is feasible within a reasonable timeframe. Despite their limitations, dengue risk maps can be powerful tools to facilitate decision making in public health, ranging from surveillance to prediction maps. The further development of this tool, useful both for research and public health applications, will depend on the acquisition and availability of diverse data of good quality with adequate requirement in term of spatial and temporal resolution.

## Electronic supplementary material

Additional file 1: Table S1: Detailed content of reviewed articles using direct extraction. (DOCX 45 KB)

Additional file 2: Table S2: Types of modeling approaches vs. types of predictors used in reviewed articles. (DOCX 18 KB)
