# Supplementary material for: Modeling tools for dengue risk mapping - a systematic review
Source: Int J Health Geogr. 2014 Dec 9;13:50. doi: 10.1186/1476-072X-13-50 (PMC4273492; doi:10.1186/1476-072X-13-50)
Supplement: Supplementary file 1 — Additional file 1: Table S1: Detailed content of reviewed articles using direct extraction. (DOCX 45 KB) [file 12942_2014_614_MOESM1_ESM.docx]

Supplementary Table S1. Detailed content of studies using direct extraction from the publications

| **ID/Author(s)/ country** | **Title** | **Research questions / Focus** | **Methods / Models** | **Key findings /Significant parameters** | **Recommendations / Dengue control** |
| --- | --- | --- | --- | --- | --- |
| **1**  Arboleda et al.  Colombia | Mapping environmental dimensions of dengue fever transmission risk in the Aburrá Valley, Colombia | Description of exploration of ecological niche dimensions and associated geographic distributions for DF cases | MaxEnt algorithm: generates predictions from incomplete sets of information based on a probabilistic framework | Ecological niche modeling showed predictive power regarding DF case distributions; In discriminating between suitable and unsuitable areas, no single variable offers clear separation. Rather, differentiation in suitability is clearly in multivariate space, and depends on complex combinations of variables.  The environment-based approaches built, offer the potential to predict transmission risk even in areas for which no sampling is available based on their environmental characteristics. | The author’s conclusion is that effective risk maps can be developed based on analyses of presence-only occurrence data.  They suggest that areas predicted by their models as suitable for DF could be considered as at-risk, and be used to guide campaigns for DF prevention in the researched municipalities. |
| **2**  Castillo et al.  Ecuador | Application of spatial analysis to the examination of dengue fever in Guayaquil, Ecuador | Elucidation of spatial distribution patterns of reported cases of dengue fever for the city of Guayaquil, Ecuador | Cluster analysis, LISA (Local Indicator of Spatial Association) | Dengue is mostly present in local hotspots. Concentrated in low income neighborhoods with limited access to public utilities  The dynamics of dengue fever transmission evolved during the study period, because from 2008-2009, clusters of disease transmission in northeast Guayaquil were found in both middle and low income areas. | Clustered areas should be considered primarily for implementation of preventive measures |
| **3**  Cordeiro et al.  Brazil | Spatial distribution of the risk of dengue fever in southeast Brazil, 2006-2007 | Estimation of the spatial distribution of dengue fever risk in an area of continuous dengue occurrence. | Population-based spatial case-control study with multinomial response with geographical location of residence as the independent variable of major interest. Logistic binomial model. Generalized additive  models (GAM). | Variables associated with increased incidence of all dengue cases in the multiple binomial regression model were: **higher larval densities, reports of mosquito bites during the day, the practice of water storage at home, low frequency of garbage collection and lack of basic sanitation. Staying at home during the day** was protective against the disease.  When cases were analyzed by categories (mild and severe) in the multinomial model, age and number of breeding sites more than 10 were significant only for the occurrence of severe cases. Spatial distribution of risks of mild and severe dengue fever differed from each other in the 2006/2007 epidemic, in the study area. | Maps help to identify sites with increased risk in densely populated cities where the risk for contracting dengue fever is greatest. Moreover, the use of generalized additive models and multinomial logistic analysis may help identify specific spatial transmission patterns. |
| **4**  de Mattos Almeida et al.  Brazil | Spatial Vulnerability to Dengue in a Brazilian Urban Area During a 7-Year Surveillance | Evaluation of the association between socioeconomic, demographic, and urban infrastructure variables and risk areas classified according to dengue occurrence and the persistence of transmission | Anova, multivariate logistics, Kruskal–Wallis test variance comparisons and multivariate regression using multinomial models. | **Low educational level (less than 4 years of schooling), low income of the head of the family (less than 2 minimum wages per household), household density, and proportion of children and elderly women** were the factors that characterized the risk areas for dengue best.  Higher dengue incidence in 26 to 32 years old in all waves and a higher incidence among women.  The difference between risk categories for variables regarding schooling level, income of family head, mean residents per household, and proportion of children and elderly women were statistically significant and confirmed the observed gradients. | The traditional indicator of mosquito infestation, based on larval surveys, was not accurate enough to estimate dengue risk. The authors state that their study may provide one way to overcome this limitation as introducing other variables to classify risk areas, especially for surveillance purposes. |
| **5**  de Melo et al.  Brazil | Dengue Fever Occurrence and Vector Detection by Larval Survey, Ovitrap and MosquiTRAP: A Space-Time Clusters Analysis | Evaluation of how different methods of *Aedes aegypti* surveillance correlate to dengue fever occurrence in a pre-selected area | Space-time clusters + cluster intersection | **MosquiTRAP and ovitrap** presented temporal and spatial intersections with dengue fever clusters, whereas **no spatial or temporal intersection was observed among larval survey** and dengue fever clusters at any analysis performed. | If entomological parameters are used for dengue risk maps, either egg or adult counts should be used; **larvae counts show no correlation.** |
| **6**  Dickin et al.  Malaysia | Developing a Vulnerability Mapping Methodology: Applying the Water-Associated Disease Index to Dengue in Malaysia | The objective of this paper is to describe and validate the WADI tool by applying it to dengue. | Spatial multiparametrical prediction model WADI (Water Asscociated Disease Index) | Model and validation dataset are significantly associated. The index output indicated high vulnerability to dengue in urban areas, especially in the capital Kuala Lumpur and surrounding region. However, in other regions, vulnerability to dengue varied throughout the year due to the influence of seasonal climate conditions, such as monsoon patterns. At altitude lower risk due to lower temperature. | The authors propose that for policy implications an aggregated value is helpful for decision makers who must otherwise draw individual conclusions about many different elements mediating transmission of water-associated diseases. |
| **7**  Flauzino et al.  Brazil | Spatial heterogeneity of dengue fever in local studies, City of Niterói, Southeastern Brazil | Analysis of the spatial and temporal occurrence of dengue fever and its association with the heterogeneity of urban environment characteristics. | Cases were grouped into five periods – two inter-epidemic periods (1998-2000 and 2003-2005) and three epidemic periods (2001, 2002 and 2006) – and analyzed using operations between layers in a geographic information system (GIS) environment. The kernel method was used to identify clusters of cases. Kulldorff’s spatial scan statistic was used to confirm these clusters statistically. | Of all cases, 57% were females. Age groups with the highest number of cases were 20-29-years (20.5%) and 30-39-years (17.7%). This pattern did not change over study years. 38% of cases occurred in the economically active age group. The hill slum sector showed only 11% of households covered by garbage collection service, the highest percentage of illiterate individuals (8.7%) and head of families with income lower than one monthly minimum wage (29.5%). Cases remained in the slum sectors. In the first epidemic year and in the inter-epidemic periods, the highest number of cases was found in the hill and flatland slum sectors; In the second and third epidemic years, in the flatland slum sector. The **economically active portion of the population was that most affected** in the study area. | Implications for dengue control can be deducted from the results. Females, certain age groups and settlement areas need more intervention/ education than others. |
| **8**  Galli & Neto  Brazil | Temporal-spatial risk model to identify areas at high-risk for occurrence of dengue fever | Applying a temporal-spatial model to assess high-risk areas for the occurrence of dengue fever. | The local indicator of spatial association was adopted to identify significant spatial clusters. The values of  the three indices were considered high in a spatial unit when their standard values were positive and the respective local indicator of spatial association values were significant. | Of all geocoded dengue fever cases, 38.1% occurred in the urban spatial units, classified as highest-risk: 19.4% in 2001-2002, 13.9% in 2002-in 2003, 2.8% in 2003-2004, 16.7% in 2004-2005, and 21.3% in 2005-2006. The **utilization of three risk measures enabled to identify higher-risk areas** for the occurrence of dengue fever, concentrated in the city’s northern region. | Even though case notification data are subject to bias, **this information is available in the health services and can lead to important conclusions, recommendations and hypotheses**. |
| **9**  Hassan et al.  Malaysia | Risk mapping of dengue in Selangor and Kuala Lumpur, Malaysia | Creation of a map of the distribution of dengue fever aiming at the identification and visualizing areas of risk | Using kriging & co-kriging, to model population density and rainfall map against the dengue rate map | Population density in itself is a good measure of risk in the author´s opinion; Supports the notion of a direct, positive correlation between population density and risk for outbreaks | With early prevention steps, such as dealing with breeding spots at places shown to receive high volumes of rainfall, the impact of outbreaks can be reduced. In areas with high population density, community-centered awareness campaigns are the best way to alert the population and make them take precautions with respect to the risk for dengue transmission. |
| **10**  Honorio et al.  Brazil | Spatial Evaluation and Modeling of Dengue Seroprevalence and Vector Density in Rio de Janeiro, Brazil | Modeling the spatial patterns of dengue seroprevalence in three neighborhoods with different socioeconomic profiles in Rio de Janeiro. Assessing the relationship between dengue seroprevalence, recent dengue infection, and vector density. | Linear models to include non- parametric smoothing terms. Generalized Additive Model (GAM). | Recent dengue infection varied from 1.3% to 14.1% among study areas. **The highest IgM seropositivity occurred in the slum, where mosquito abundance was the lowest, but household conditions were the best for promoting contact between hosts and vectors**. Only 23.3% of infections were symptomatic, suggesting that even during severe epidemics, silent circulation of the virus is highly prevalent. Recent dengue infection in residences located in areas with low mosquito densities**, suggesting that infection took place out of the residence, either in other premises or outdoors. No association between recent dengue infection and household’s high mosquito abundanc**e was observed in the sample. Significantly higher risk patches close to the areas with large human movement, suggests that humans may be responsible for virus inflow to small neighborhoods in Rio de Janeiro | The variation in spatial seroprevalence patterns inside the neighborhoods, with significantly higher risk patches close to the areas with large human movement, suggests that **humans may be responsible for virus inflow to small neighborhoods** in Rio de Janeiro. |
| **11**  Hu et al.  Australia | Spatial Patterns and Socioecological Drivers of Dengue Fever Transmission in Queensland, Australia | Examination of the impact of socioecological factors on the transmission of DF and assess potential predictors of locally acquired and overseas-acquired cases of DF in Queensland, Australia. | Bayesian spatial conditional autoregressive model Separate Poisson regression models were developed in a Bayesian frame- work for locally acquired and overseas-acquired cases, | Results showed an increase in locally acquired DF of 6% in association with a 1-mm increase in average monthly rainfall and a 1°C increase in average monthly maximum temperature between 2002 and 2005, respectively. By contrast, overseas-acquired DF cases increased by 1% in association with a 1-mm increase in average monthly rainfall and a 1-unit increase in average socioeconomic index, respectively. Higher average SEIFA score (indicating LGAs with higher average socio- economic status) is associated with an increase in the number of overseas-acquired DF cases. | An early warning system for DF based on a Bayesian spatial model would facilitate the early identification of impending epidemics, which could lead to a more rapid response than is possible currently, thereby reducing the magnitude and health and economic impact of epidemics. |
| **12**  Jeefoo et al.  Thailand | Spatio-temporal diffusion pattern and hotspot detection of dengue in Chachoengsao province, Thailand. | Understanding of temporal dynamics and spatial correlations of DF | Global Moran´s I statistic to identify spatial patterns, Hotspot detection with LISA method (Local Indicators of Spatial Association), Spatial Analysis | The results revealed spatial diffusion patterns during the years 1999–2007 representing spatially clustered patterns with significant differences by village. Villages on the urban fringe reported higher incidences. The space and time of the cases showed outbreak movement and spread patterns that could be related to entomologic and epidemiologic factors  The age group of 13-24 years showed 43% of cases; temporal distribution depending on rainfalls, showing a peak in June. | Dengue spatio-temporal diffusion patterns and hotspot detection may provide useful information to support public health officers to control and predict dengue spread over critical hotspot areas only rather than for a whole province. its significance.  **Gender and age groups vulnerability is also an interesting outcome of the study**. |
| **13**  Khormi & Kumar  Saudi Arabia | Modeling dengue fever risk based on socioeconomic parameters, nationality and age groups: GIS and remote sensing based case study | Creation of a predictive risk model of people likely to be infected by dengue fever | Geographically weighted regression (GWR), | **Neighborhood quality** can be a good determinant parameter for predicting areas at high risk; DF is mostly prevalent in districts that have low neighborhood quality, high population density, and a high percentage of non-Saudis, while the districts with low risk of DF have high neighborhood quality, low population density, and a high percentage of Saudis. Saudis more at risk than non-Saudis in 2007 and 2008, non-Saudis more at risk from 2010 onwards; Age range 16-30 years with highest infection rates, the above 60 year old with the lowest rates. Increased neighborhood quality and low population density correlate both with low dengue risk. | High risk areas should be under constant monitoring and treatment; adult age group needs intensive educational program. |
| **14**  Khormi et al.  Saudi Arabia | Modeling spatio-temporal risk changes in the incidence of dengue fever in Saudi Arabia: a geographical information system case study | Modeling of annual hotspots for dengue cases and the vector mosquitoes. Use of GIS to demonstrate the dengue fever risk on a monthly basis in Jeddah, Saudi Arabia for improved surveillance and monitoring. Visualization of the progress of epidemics. | Getis-Ord Gi, frequency index | The average monthly risk over a 5 year period shows dengue hotspots clustered in central Jeddah, while northern and southern districts show significantly lower risk. **.** | The application of average weekly frequency indices makes it possible to recognize monthly disease patterns, which facilitates the assessment of the strengths and weaknesses of current control measures. |
| **15**  Khormi & Kumar  Saudi Arabia | Assessing the risk for dengue fever based on socioeconomic and environmental variables in a geographical information system environment | Developing of a system which uses current DF and Aedes aegypti data to assess risk areas, furthermore the inclusion of environmental and socioeconomic parameters. Model focus lays more on current risk rather than on future developments. | Getis-Ord Gi, spatial patterns | Areas with a low risk for DF had a low mean population density (2,107 per km2), while areas of medium risk had a medium mean population density (12,880 per km2) and areas of high risk had the highest mean population density (19,728). The ratio between Saudi and expatriate population was found to be 65% to 35% in the low-risk areas, 49%-51% in the areas of medium risk, it was 47%-53% in areas of high risk ; people are highly mobile and are often outside their districts for work or visiting relatives and friends. **From 2006 to 2008, the prevalence of DF and mosquito abundance was strongly associated**.  **Most of the victims were expatriates**, around 66% in 2009 and around 77% in 2010…inhabiting low-quality neighbourhoods, which favour  mosquito breeding, in districts with high A. aegypti densities.; limited access to water, high population density high building density, and low neighborhood quality. | The models developed in the study comprise an approach to DF control and prevention that can be utilized for control management and improved surveillance. Conclusions and recommendations: (i) The hotspot model based on annual data provides overviews of high-impact areas by DF, while spatio-temporal risk modelling, based on monthly and weekly frequency indices, identifies rapid changes. (ii) Risk models of DF, based on a combination of environmental and socioeconomic variables, can help to define the causes behind the prevalence of the disease. (iii) Elimination of mosquito breeding sites and providing vulnerable populations with window screens, safe water containers and better access to water supplies are likely to lower DF transmission. (iv) The overall model can be used by the decision makers in Jeddah municipality for prioritizing when carrying out the major infrastructure projects planned in Jeddah. |
| **16**  Lowe et al.  Brazil | Spatio-temporal modeling of climate-sensitive disease risk: Towards an early warning system for dengue in Brazil | Exploring the potential for using seasonal climate forecasts in developing an early warning system for dengue fever epidemics | Negative binomial model allowing for overdispersion effects. Spatio-temporal hierarchical model (GLMM—generalised linear mixed model) implemented via a Bayesian framework using Markov Chain Monte Carlo (MCMC). | **Climatic covariates play a statistically significant role** in the transmission of dengue fever. Although climate information alone does not account for a large proportion of the overall variation in dengue cases in Brazil, spatio-temporal climate information with the addition of spatio-temporal random effects do account for some of this variability, particularly for the 2008 peak dengue season, when a serious epidemic occurred. Dengue predictions are found to be enhanced both spatially and temporally when using the GLMM and the Bayesian framework allows posterior predictive distributions for dengue cases to be derived, which can be useful for developing a dengue alert system. Using this model, the authors conclude that seasonal climate forecasts could have potential value in helping to predict dengue incidence months in advance of an epidemic in South East Brazil. | The **spatio- temporal models** offers an opportunity to balance global climate variables and local responses, e.g. the influence of ENSO on dengue incidence is likely to occur unequally across the region due to particular socio-economic local conditions. Further capacity of the GLMM is the ability to address specific public health issues in terms of probabilities. The authors propose possible model extensions to other regions in the world, applicable to other climate-sensitive infectious diseases. |
| **17**  Machado-Machado  Mexico | Empirical mapping of suitability to dengue fever in Mexico using species distribution modeling | Development of dengue suitability maps based on climatic, environmental and socioeconomic factors. | Maxent algorithm, species distribution modeling technique | Lowland and coastal areas show lower dengue risk. The minimum temperature of the coldest month and mean temperature of the coldest quarter were the variables with the most useful information | The modeling approach points out a country scale model for dengue fever suitability, based on numerous, mainly environmental variables. Due to its scale, public health recommendations will be equally available only up to this level. |
| **18**  Peterson et al.  Mexico | Time-specific ecological niche modeling predicts spatial dynamics of vector insects and human dengue cases | Development and test of the hypothesis that the details of spatial and temporal dynamics of vector populations and consequent disease outbreaks can be predicted via ecological niche modeling | Ecological niche models using the Genetic Algorithm for Rule-set Prediction(GARP); random walk. Coverages included elevation, slope, aspect and tendency to pool  water (topographic index). | Prediction of spatial and temporal vector abundance based on environmental variables was possible for large parts during the study years. **Vector activity coincides with human DF cases.**  Slope, topographic index and aspect were statistically insignificant in determining model  predictivity. | Genetic Algorithm seems well suited for ecological niche modeling.  Authors propose linking with remotely sensed data to predict key areas of DF transmission. |
| **19**  Porcasi et al.  Argentina | An operative dengue risk stratification system in Argentina based on geospatial technology | Creation of maps for risk stratification at national and urban level | Maximum  Entropy method and logistic regression based on the Wald forward stepwise method. | The developed DRS (dengue risk stratification system) informatics platform allows operational flexibility and enables the visualization of intermediate products of the risk assessment process evaluating different aspects of vector control and surveillance activities in detail. The general architecture makes the platform applicable for risk mapping of many different vector-borne diseases. | Software product does not require professional specialization and provides a user-friendly tool for all end-users and decision makers.  When the study was published 48 localities were sharing the risk stratification system. |
| **20**  Rotela et al.  Argentina | Space-time analysis of the dengue spreading dynamics in the 2004 Tartagal outbreak, Northern Argentina | Generation of a risk maps on the basis of the 2004 dengue outbreak in Tartagal using GIS and remote sensing. Assess the effect of the national dengue prevention program. | Analysis of spatio–temporal clustering of DF cases using the Knox test concept. Fourier harmonic analysis. | Differences in of DF occurrence amongst different age groups. Incidence map of simulated dengue cases shows clusters of agglomeration. | A first step to implement daily GIS dengue maps of incidence evolution and to develop prediction models based on environmental and epidemiological data. These tools could help decision-makers to improve health system responses and prevention measures related to vector control. |
| **21**  Shafie  Malaysia | Evaluation of the Spatial Risk Factors for High Incidence of Dengue Fever and Dengue Hemorrhagic Fever Using GIS Application | Developing spatial models for areas with DF risk and prediction of DF and DHF in each area | Stepwise logistic regression | 10 of 16 tested variables show significant influence in the logistic regression for DF and DHF incidence probability. **The significant variables are proximity to cemeteries, proximity to swamp and forest areas, proximity to river bank areas, proximity to public infrastructure areas, proximity to parks, proximity to school areas, proximity to industrial areas, proximity to hospital areas, proximity to government institution areas and population density**. | The derived maps could provide useful information to health authorities and could assist in focusing and implementing control and preventive activities to monitor and control the incidence of dengue precisely and effectively, especially in the event when there is no report on dengue cases |
| **22**  Sriprom et al.  Thailand | Monthly district level risk of dengue occurrences in Sakon Nakhon Province, Thailand | Provision of a smooth distribution of dengue virus infection incidence and identify locations (at the district level) and periods of the year at risk of occurrence of DVI cases in Sakon Nakhon Province, Thailand | Generalized Linear Model (GLM), spatio-temporal | The GLM indicates that the number of cases **increases significantly with the number of children of 0–4 years old, while it decreases significantly with both the proportion of villages with primary schools and per capita number of public small water wells**; monthly minimum temperature will likely increase the DVI incidences of districts and expand the transmission period in weakly endemic (less populated) districts. | Where and when the dengue virus circulation occurs are useful information that can be used by provincial health officials in designing and targeting control measures; : 1) start the surveillance system earlier in April when weather conditions are favorable , 2) prioritize the surveillance efforts and control measures from most to least populated districts, and 3) promote the increase of the number of private wells |
| **23**  Wen et al.  Taiwan | Spatial mapping of temporal risk characteristics to improve environmental health risk identification: a case study of a dengue epidemic in Taiwan | Development of a spatio– temporal risk model for mapping geographic distribution of uneven events with temporally defined indices to improve health risks identifications with focus on three temporal risk characteristics across geographic space: (1) frequency of occurrence of uneven cases, (2) persistence of cases, (3) significance of case occurrence in consecutive periods | 3 temporal indices: frequency, duration, intensity; The local indicator of spatial autocorrelation (LISA) was adopted as the spatial risk index to identify significant spatial clusters | The range of the duration index has the largest variation; About 78.4 % of Li(s) (425/542) are identified as type H with all three indices categorized as low, indicating that the DF cases have no significant temporal patterns in those areas | Improvement of case cluster detection. Exploration of temporal characteristics. Methods applicable in the modeling for other infectious diseases. |
| **24**  Wen et al.  Taiwan | Spatial-temporal patterns of dengue in areas at risk of dengue hemorrhagic fever in Kaohsiung, Taiwan, 2002 | Examination whether spatial–temporal patterns of dengue can be used to identify areas at risk of dengue hemorrhagic fever | 3 temporal indices: frequency, duration, intensity; spatial autocorrelation, 5risk categories: extreme, high, moderate, mild, low | Two significant clusters of high incidence were found; in some districts the 3 indices did not correlate very well with the cumulative incidence. 3 temporal indices, 8 risk types, 5 risk levels; With regard to DEN/POP, areas with the Hi-ODI risk type showed the greatest cumulative incidence | The study provides public health authorities with tool to differentiate risk patterns of a dengue epidemic using three additional temporal indices rather than relying on annual cumulative incidence alone, so that high-risk areas can be identified early in the epidemic based on their integrated spatial–temporal profiles. The method also directs broader perspectives on the temporal risks within the epidemic curves and emphasizes surveillance efforts at the tail end of an epidemic period. Generalization and appliance in other infectious disease might be possible. |
| **25**  Wu et al.  Taiwan | Higher temperature and urbanization affect the spatial patterns of dengue fever transmission in subtropical Taiwan | Development of risk maps by examination of the effect of temperature and other environmental factors affect dengue fever distributions. Forecasting areas with potential risk for dengue fever endemics with predicted climatic change in Taiwan. Exploration of relationships between cumulative incidence of dengue fever, climatic and non-climatic factors | Spatial empirical Bayes smoothing method. Logistic regressions. Kernel estimation.  Included 11 factors for each township, including average population density, income,  percentage of service and agriculture occupancy, home ownerships, household overcrowding, aborigine, elders, elders living alone, disability, and numbers of clinics. Recovery rate of household vectors (based on Breteau Index) | **The imported incidence, the household**  **vectors recovery rate, annual rainfall, higher elder and aborigine population were not identified as the significant variables.**  Numbers of months with average temperature higher than 18 °C per year and degree of urbanization were found to be associated with increased risk of dengue fever incidence at township level. With every 1 °C increase of monthly average temperature, the total population at risk for dengue fever transmission would increase by 1.95 times (from3, 966,173 to 7,748,267). A highly-suggested warmer trend, with a statistical model, across the Taiwan Island is predicted to result in a sizable increase in population and geographical areas at higher risk for dengue fever epidemics. | The derived risk map could be adopted to formulate the strategy for the national dengue fever control program in the future |
| **26**  Yu et al.  Taiwan | A spatio-temporal climate-based model of early dengue fever warning in southern Taiwan | Generation of a spatio-temporal early warning system model (EWS) of DF based on the stochastic Bayesian Maximum Entropy (BME) method and quantitatively assess the spatio-temporal epidemic dependence to generate informative maps of the epidemic variables of interest. Parameters: climate variables, Breteau index, health datasets | Bayesian Maximum Entropy (BME) analysis Stochastic Bayesian maximum entropy; stepwise selection procedure Poisson (Temperature (avg, min, max); precipitation; SOI, Breteau index)  Spatio-temporal dependence functions representing DF incidences space.  Time trend estimated by a nonparametric kernel smoothing method | Climate-based Poisson regression model can provide similar general temporal pattern of dengue incidences but does not fulfill the satisfactory predictions regarding the magnitude of the DF outbreak. Predicts the serious outbreak cases but not the much less serious ones. The high DF risk extends to an area of about 20 km radius around its outbreak foci.  Once a DF epidemic occurs at a certain location, its neighborhood will be under a higher infection risk for about 20 weeks (the risk is highest within the first 2 weeks).  The selected temporal lags range of 8–12 weeks. DF incidences are negatively associated with the average and maximum temperatures and the DF incidences at the selected temporal lags. | The results show that the DF outbreaks in the study area are highly influenced by climatic conditions.  During every week of the epidemic, the present analysis correctly detected the spatial locations of the actual DF hot-spots.  The analysis can provide the required ‘‘one week-ahead’’ outbreak warnings based on spatio-temporal predictions of DF distributions. |
