# Supplementary material for: Modeling tools for dengue risk mapping - a systematic review
Source: Int J Health Geogr. 2014 Dec 9;13:50. doi: 10.1186/1476-072X-13-50 (PMC4273492; doi:10.1186/1476-072X-13-50)
Supplement: Supplementary file 2 — Additional file 2: Table S2: Types of modeling approaches vs. types of predictors used in reviewed articles. (DOCX 18 KB) [file 12942_2014_614_MOESM2_ESM.docx]

**Supplementary table S2.** Publications categorized by type of modeling approaches and type of parameters

|  | **Types of parameters** | **Population** | **Demogr.** | **Socio-Economic** | **Climato-logical** | **Environ-mental** | **Entomo-logical** | **Remote sensing** | **Temporal** | **Total nb articles** | | |
| --- | --- | --- | --- | --- | --- | --- | --- | --- | --- | --- | --- | --- |
|  | **Types of modeling approaches** |  |  | (ID of reviewed articles) | | | | | | | N |  |
| 1 | Spatial analysis of case clusters/hotspots | 2,7,8,10,12,14,15 | 2,7,8,10,12,14,15,20 | 7,10 | 5,12,26 | 15,20,26 | 5,10,15,26 | 10,12,15,20 | 5,7,8,12,14,15,20,26 | 10 | | |
| 2 | Spatial autocorrelation measures | 2,12,14,15 | 2,12,14,15,20 |  | 5,12 | 15,20 | 5,15 | 12,15,20 | 5,12,14,15,20 | 6 | | |
| 3 | logistic regression and multinomial models | 3,4,19,21,25 | 3,4,25 | 3,4,19,25 | 19,21,25,26 | 19,21,26 | 3,25,26 | 3,19 | 26 | 6 | | |
| 4 | General additive model (GAM)/Generalized linear models (GLM)/ Generalized linear mixed models (GLMM) | 3,10,11,16, 22 | 3,10,11,22 | 3,10,11,16,22 | 11,16,22 | 16 | 3,10, | 3,10,11 | 16, 22 | 5 | | |
| 5 | Kernel estimation | 7,12,25 | 7,12,25 | 7,25 | 12,25,26 | 26 | 25,26 | 12 | 7,12,26 | 4 | | |
| 6 | Environmental niche modeling/ Species distribution modeling (Suitability) | 17 |  | 17 | 17 | 1,18 | 18 | 1,18 | 18 | 3 | | |
| 7 | Maximum Entropy (MaxEnt) | 17,19 |  | 17,19 | 17,19,26 | 1,19,26 | 26 | 1,19 | 26 | 4 | | |
| 8 | Geographically weighted regression | 13 |  | 13 |  |  |  | 13 |  | 1 | | |
| 9 | Kriging and ko-kriging | 9 |  |  | 9 |  |  |  |  | 1 | | |
| 10 | Knox test concept (space- & time-distance) |  | 20 |  |  | 20 |  | 20 | 20 | 1 | | |
| 11 | Temporal indices (occurrence, duration, intensity) | 8,23,24 | 8 |  |  |  |  |  | 8,23,24 | 3 | | |
| 12 | Water-associated disease index -WADI (Vulnerability) | 6 | 6 | 6 | 6 | 6 |  | 6 |  | 1 | | |
